# Impact of tumor volume enlargement after induction chemotherapy on subsequent radiotherapy in locally advanced nasopharyngeal carcinoma: A propensity‐score matching analysis

**DOI:** 10.1002/cam4.3494

**Published:** 2020-10-06

**Authors:** Shan Li, Liangfang Shen

**Affiliations:** ^1^ Department of Oncology Xiangya Hospital Central South University Changsha Hunan Province China

**Keywords:** dosimetry, induction chemotherapy, nasopharyngeal carcinoma, propensity score matching, tumor volume

## Abstract

A small proportion of nasopharyngeal carcinoma (NPC) patients show resistance to induction chemotherapy (IC). This study sought to investigate the impact of tumor volume enlargement after IC on the dosimetric parameters of subsequent radiotherapy. The records of a total of 240 locally advanced NPC patients who received IC followed by concurrent chemoradiotherapy were retrospectively reviewed. Patients with a tumor volume enlargement of ≥10% and patients with a tumor volume reduction of ≥10% after induction chemotherapy were classified as the enlargement group and the control group, respectively. The dosimetric parameters of the planning target volumes (PTVs) and the organs at risk (OARs) were compared between the matched groups after propensity score matching (PSM). For the gross tumor volume of nasopharynx (GTVnx), 21 patients and 127 patients were classified as the enlargement group and the control group, respectively. After matching, 20 sub‐pairs of 40 patients were generated in the post‐PSM cohort. The GTVnx enlargement group exhibited no significant disadvantages in all of the dosimetric parameters, except in the planning organ‐at‐risk volume (PRV) of contralateral lens (Dmax, 722 cGy vs. 634 cGy, *p* = 0.041). For the gross tumor volume of lymph nodes (GTVnd), 44 patients and 144 patients were classified as the enlargement group and the control group, respectively. After matching, 39 sub‐pairs of 78 patients were generated in the post‐PSM cohort. The GTVnd enlargement group exhibited no significant disadvantages in all of the dosimetric parameters. Univariate and multivariate analyses showed that the enlargement of GTVnx and the enlargement of GTVnd were not independently associated with any of the dosimetric parameters. A tumor volume enlargement of ≥10% in GTVnx or GTVnd after induction chemotherapy has no significant impact on the dosimetric parameters of subsequent radiotherapy in locally advanced NPC.

## Graphical abstract

A small proportion of nasopharyngeal carcinoma patients show resistance to induction chemotherapy. A tumor volume enlargement of ≥ 10% in the primary tumor or the lymph nodes after induction chemotherapy has no significant impact on the dosimetric parameters of subsequent radiotherapy.

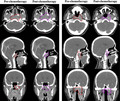

## 1 INTRODUCTION

Nasopharyngeal carcinoma (NPC) is a common type of head and neck cancer, and radiotherapy is its principle treatment method due to the complicated anatomical location of the cancer and its high sensitivity to radiation.^1^, ^2^ Recently, induction chemotherapy (IC) followed by concurrent chemoradiotherapy has become a standard treatment strategy for locally advanced NPC according to the National Comprehensive Cancer Network (NCCN) guidelines (version 1.2020). Several well‐designed randomized controlled studies have shown that IC can improve the survival outcomes of NPC patients.^3^, ^4^, ^5^ However, it is noteworthy that although most NPC patients are sensitive to chemotherapy, a small proportion of patients show resistance to IC, which has a negative influence on patient survival.^6^, ^7^

In addition to the impact on patient survival, another potential consequence of tumor progression after IC is the influence on subsequent radiotherapy. The location of NPC is surrounded by many important structures, such as the brainstem, spinal cord, and optic nerves.^8^, ^9^ An increase in tumor volume after IC may have a significant influence on the dosimetric parameters of the subsequent radiotherapy plan. However, no publications have addressed this problem thus far. Therefore, we conducted this retrospective study to compare the radiotherapy plans of patients with tumor volume enlargement and patients with tumor volume reduction after IC using the propensity score matching (PSM) method.

## 2 MATERIAL AND METHODS

### 2.1 Patient selection

A total of 240 NPC patients were selected according to the following criteria: (a) locally advanced NPC (T1‐2N1‐3M0 or T3‐4N0‐3M0, according to the AJCC 8th staging system) with pathology confirmation; (b) treated with IC followed by concurrent chemoradiotherapy at our hospital from 2016 to 2019; (c) contrast‐enhanced simulation CT and simulation MRI were performed before and after IC; and (4) the radiotherapy plan was available for review. Written informed consent was obtained from each subject.

### 2.2 Induction chemotherapy

All of the patients received IC with docetaxel plus cisplatin (docetaxel 75 mg/m^2^ day 1, cisplatin 75 mg/m^2^ day 2) for two or three cycles. The chemotherapy cycle was repeated every 21 days. Adequate bone marrow function, liver function, and renal function were required before the start of each chemotherapy cycle. All of the patients underwent contrast‐enhanced simulation CT and simulation MRI at a 3‐mm slice thickness with immobilization devices before IC. The gross tumor volume of nasopharynx (pre‐IC GTVnx) and the gross tumor volume of lymph nodes (pre‐IC GTVnd) were contoured with CT and MRI fusion images by a medical team consisting of radiation oncologists and radiologists.

### 2.3 Concurrent chemoradiotherapy

Radiotherapy was delivered 3 weeks after the last cycle of IC, concurrent with cisplatin 80–100 mg/m^2^ every three weeks or 30–40 mg/m^2^ every week.^10^ Contrast‐enhanced simulation CT and simulation MRI at a 3‐mm slice thickness with immobilization devices were performed again for the preparation of radiotherapy. The gross tumor volume of nasopharynx (post‐IC GTVnx) and the gross tumor volume of lymph nodes (post‐IC GTVnd) were contoured again by the same medical team.

The target volumes in the radiotherapy plan included the final GTVnx, the final GTVnd, the clinical target volume 1 (CTV1), and the clinical target volume 2 (CTV2) according to the recommendation of the international guideline for the delineation of the clinical target volumes for NPC.^11^ The final GTVnx was defined as the summation of the pre‐IC GTVnx and the post‐IC GTVnx, which included all of the areas involved by the primary tumor before and after IC. The final GTVnd was defined as the post‐IC GTVnd only. CTV1 and CTV2 were defined as the high‐risk volume and the low‐risk volume, respectively.

An expansion of 3–5 mm around the final GTVnx, the final GTVnd, CTV1, and CTV2 was adopted to generate the corresponding planning target volumes (PGTVnx, PGTVnd, PTV1, and PTV2). The prescription doses delivered to PGTVnx, PGTVnd, PTV1, and PTV2 were 70.4 Gy (2.2 Gy per fraction), 70.4 Gy (2.2 Gy per fraction), 60.8 Gy (1.9 Gy per fraction), and 54 Gy (1.8 Gy per fraction), respectively. The organs at risk (OARs) included the spinal cord, brain stem, optic chiasm, optic nerves, lenses, temporal lobes, parotid glands, and pituitary. Additionally, an expansion of the brain stem, spinal cord, and lens by 1, 5, and 5 mm, respectively, was adopted to generate the corresponding planning organ‐at‐risk volumes (PRVs).

The radiotherapy planning techniques consisted of conventional intensity‐modulated radiation therapy (IMRT) and tomotherapy. The conventional IMRT plans, which included the volumetric‐modulated arc therapy and the step‐and‐shoot IMRT, were generated with the Eclipse treatment planning system (Eclipse version 11.3, Varian Medical Systems). The tomotherapy plans were generated with the TomoTherapy Planning Workstation (TomoHD version 2.0.7, Accuracy Inc.).

### 2.4 Dosimetric comparisons

Patients with a tumor volume enlargement of ≥10% and patients with a tumor volume reduction of ≥10% after IC were classified as the enlargement group and the control group, respectively. PSM was adopted to control the balance between the enlargement group and its control group. Matching covariates in the score scale included T stage, N stage, plan type, pretreatment volume of GTVnx, and pretreatment volume of GTVnd. For the PTVs, the minimum coverage dose of 95% of the target (D95) was selected as the dosimetric parameter for comparisons in the post‐PSM cohort. For the OARs, the maximum dose (Dmax) was adopted to evaluate the dosimetric differences of the brainstem, brainstem PRV, spinal cord, spinal cord PRV, optic chiasm, optic nerve, lens PRV, and pituitary between the matched groups in the post‐PSM cohort. In addition, the relative volume receiving over 30 Gy (V30 Gy) and the relative volume receiving over 60 Gy (V60 Gy) were selected to evaluate the dosimetry of the parotid glands and temporal lobes, respectively.

### 2.5 Statistical analyses

All of the statistical analyses were performed with SPSS (version 25, IBM SPSS Statistics). The comparisons of baseline characteristics between the enlargement group and the control group were made with the independent *t* test and chi‐square test. Dosimetric comparisons between the matched groups in the post‐PSM cohort were conducted with the independent *t* test. Univariate and multivariate analyses of dosimetric parameters were performed with the linear regression model. The variants, which showed an *α* < 0.1 in the univariate analysis, were enrolled in the multivariate analysis.

## 3 RESULTS

### 3.1 Impact of GTVnx enlargement after IC on the dosimetric parameters of subsequent radiotherapy

**FIGURE 1:**
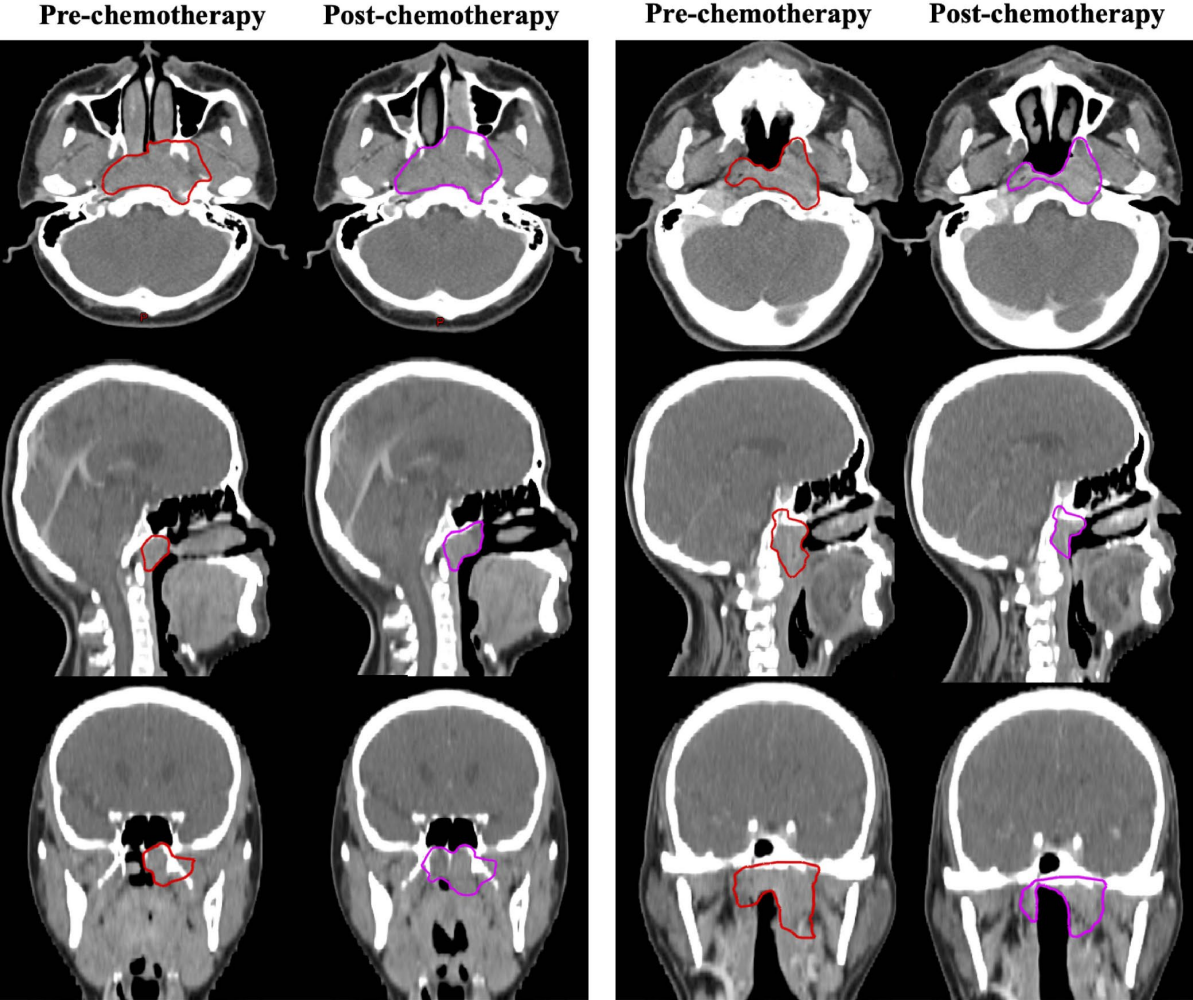
A typical case of GTVnx enlargement (left) and its matched control case(right) in the post‐PSM cohort. The red lines represent the contours of GTVnx before induction chemotherapy. The purple lines represent the contours of GTVnx after induction chemotherapy. (GTVnx = the gross tumor volume of nasopharynx)

For GTVnx, 21 patients and 127 patients were classified as the enlargement group and the control group, respectively. After matching, 20 sub‐pairs of 40 patients were generated in the post‐PSM cohort. A mean volume enlargement of 20.2% was observed in the enlargement group, and a mean volume reduction of 27.1% was observed in the matched control group. Figure 1 shows a typical case of GTVnx enlargement and its matched case in the control group. Table 1 shows the comparisons of baseline characteristics between the GTVnx enlargement group and the control group in the pre‐ and post‐PSM cohorts. As shown in Table 2, the enlargement group exhibited no significant disadvantages in all of the dosimetric parameters compared with the matched control group, except in the contralateral lens PRV (Dmax, 722 cGy vs. 634 cGy, *p* = 0.041).

**TABLE 1:** Comparisons of baseline characteristics between the GTVnx enlargement group and its control group in the pre‐ and post‐PSM cohorts

	Pre‐PSM			Post‐PSM		
	Control group (N = 127)	Enlargement group (N = 21)	*p*	Control group (N = 20)	Enlargement group (N = 20)	*p*
T stage			0.014			0.674
T1	6	4		3	3	
T2	15	5		5	5	
T3	66	5		8	5	
T4	40	7		4	7	
N stage			0.138			0.796
N0	2	0		0	0	
N1	32	5		3	5	
N2	55	14		14	13	
N3	38	2		3	2	
Plan type			0.037			1.00
Tomotherapy	99	21		19	20	
Conventional IMRT	28	0		1	0	
Pretreatment GTVnx volume (cm^3^)	43.85 ± 24.87	36.58 ± 26.92	0.221	38.64 ± 30.55	37.84 ± 26.97	0.931
Pretreatment GTVnd volume (cm^3^)	25.92 ± 25.89	18.93 ± 17.17	0.235	21.80 ± 20.49	19.29 ± 17.54	0.680

Abbreviation: PSM, propensity score matching.

**TABLE 2:** Comparisons of dosimetric parameters between the GTVnx enlargement group and its matched control group in the post‐PSM cohort

Parameters	Group	Mean	SD	*p*
PGTVnx_D95 (cGy)	Control group	7037	45	0.755
	Enlargement group	7033	34	
PGTVnd_D95 (cGy)	Control group	7090	71	0.807
	Enlargement group	7095	74	
PTV1_D95 (cGy)	Control group	6223	64	0.452
	Enlargement group	6237	58	
PTV2_D95 (cGy)	Control group	5619	180	0.467
	Enlargement group	5652	86	
Spinal cord_Dmax (cGy)	Control group	3254	265	0.617
	Enlargement group	3297	269	
Spinal cord PRV_Dmax (cGy)	Control group	4021	464	0.685
	Enlargement group	4075	357	
Brainstem_Dmax (cGy)	Control group	5071	285	0.079
	Enlargement group	5203	161	
Brainstem PRV_Dmax (cGy)	Control group	5639	342	0.176
	Enlargement group	5765	222	
Optic chiasm_Dmax (cGy)	Control group	4875	1398	0.132
	Enlargement group	5465	990	
Optic nerve I_Dmax (cGy)	Control group	5405	1073	0.376
	Enlargement group	5674	811	
Optic nerve C_Dmax (cGy)	Control group	5136	1082	0.546
	Enlargement group	5305	608	
Lens PRV I_Dmax (cGy)	Control group	697	185	0.270
	Enlargement group	762	183	
Lens PRV C_Dmax (cGy)	Control group	634	131	0.041
	Enlargement group	722	132	
Pituitary_Dmax (cGy)	Control group	6118	876	0.650
	Enlargement group	6242	824	
Temporal lobe I_V60 Gy (%)	Control group	5.26	4.88	0.479
	Enlargement group	6.43	5.49	
Temporal lobe C_V60 Gy (%)	Control group	2.25	1.96	0.921
	Enlargement group	2.19	1.63	
Parotid gland I_V30 Gy (%)	Control group	52.37	14.27	0.648
	Enlargement group	54.52	15.22	
Parotid gland C_V30 Gy (%)	Control group	47.94	13.14	0.310
	Enlargement group	52.55	15.13	

Abbreviations: C, contralateral; *D*_95_, the minimum dose delivered to 95% of the target; *D*_max_, maximum dose; I, ipsilateral; PSM, ropensity score matching; V30 Gy, the relative volume of the structure receiving over 30 Gy; V60 Gy, the relative volume of the structure receiving over 60 Gy.

### 3.2 Impact of GTVnd enlargement after IC on the dosimetric parameters of subsequent radiotherapy

**FIGURE 2:**
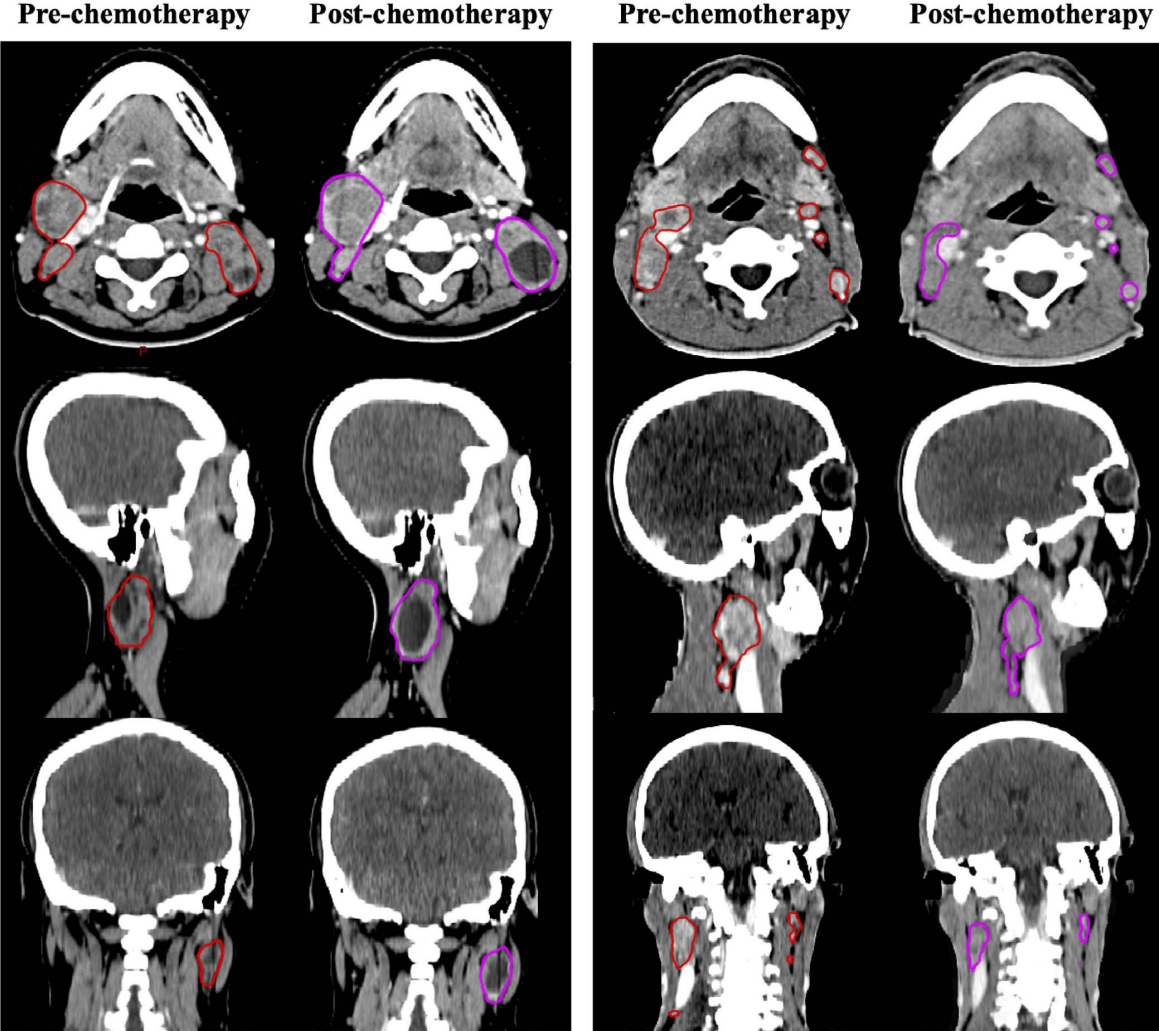
A typical case of GTVnd enlargement (left) and its matched control case(right) in the post‐PSM cohort. The red lines represent the contours of GTVnd before induction chemotherapy. The purple lines represent the contours of GTVnd after induction chemotherapy. (GTVnd =the gross tumor volume of lymph nodes)

For GTVnd, 44 patients and 144 patients were classified as the enlargement group and the control group, respectively. After matching, 39 sub‐pairs of 78 patients were generated in the post‐PSM cohort. A mean volume enlargement of 50.6% was observed in the enlargement group, and a mean volume reduction of 40.1% was observed in the matched control group. Figure 2 shows a typical case of GTVnd enlargement and its matched case in the control group. Table 3 shows the comparisons of baseline characteristics between the GTVnd enlargement group and the control group in the pre‐ and post‐PSM cohorts. As shown in Table 4, the enlargement group exhibited no significant disadvantages in all of the dosimetric parameters, compared with the matched control group.

**TABLE 3:** Comparisons of baseline characteristics between the GTVnd enlargement group and its control group in the pre‐ and post‐PSM cohorts

	Pre‐PSM			Post‐PSM		
	Control group (N = 144)	Enlargement group (N = 44)	*p*	Control group (N = 39)	Enlargement group (N = 39)	*p*
T stage			0.205			0.657
T1	10	2		3	2	
T2	22	6		3	6	
T3	74	17		19	15	
T4	38	19		14	16	
N stage			0.000			0.970
N0	0	0		0	0	
N1	23	19		15	14	
N2	68	20		19	20	
N3	53	5		5	5	
Plan type			0.064			1.000
Tomotherapy	31	4		34	35	
Conventional IMRT	113	40		5	4	
Pretreatment GTVnx volume (cm^3^)	39.79 ± 23.63	47.82 ± 28.83	0.063	46.09 ± 28.49	46.35 ± 27.75	0.967
Pretreatment GTVnd volume (cm^3^)	30.37 ± 26.09	12.52 ± 18.30	0.000	13.99 ± 12.79	13.74 ± 19.12	0.946

Abbreviation: PSM, propensity score matching.

**TABLE 4:** Comparisons of dosimetric parameters between the GTVnd enlargement group and its matched control group in the post‐PSM cohort

Parameters	Group	Mean	SD	*p*
PGTVnx_D95 (cGy)	Control group	7033	41	0.223
	Enlargement group	7006	132	
PGTVnd_D95 (cGy)	Control group	7108	66	0.519
	Enlargement group	7098	75	
PTV1_D95 (cGy)	Control group	6245	89	0.967
	Enlargement group	6244	98	
PTV2_D95 (cGy)	Control group	5591	155	0.058
	Enlargement group	5648	105	
Spinal cord_Dmax (cGy)	Control group	3335	363	0.792
	Enlargement group	3315	294	
Spinal cord PRV_Dmax (cGy)	Control group	4129	507	0.784
	Enlargement group	4156	314	
Brainstem_Dmax (cGy)	Control group	5166	236	0.171
	Enlargement group	5232	186	
Brainstem PRV_Dmax (cGy)	Control group	5752	316	0.152
	Enlargement group	5843	233	
Optic chiasm_Dmax (cGy)	Control group	5505	1270	0.995
	Enlargement group	5503	1338	
Optic nerve I_Dmax (cGy)	Control group	5761	1129	0.948
	Enlargement group	5778	1244	
Optic nerve C_Dmax (cGy)	Control group	5453	1130	0.744
	Enlargement group	5371	1061	
Lens PRV I_Dmax (cGy)	Control group	940	871	0.481
	Enlargement group	835	317	
Lens PRV C_Dmax (cGy)	Control group	782	267	0.381
	Enlargement group	739	141	
Pituitary_Dmax (cGy)	Control group	6402	772	0.975
	Enlargement group	6409	912	
Temporal lobe I_V60 Gy (%)	Control group	7.18	5.74	0.662
	Enlargement group	7.91	8.70	
Temporal lobe C_V60 Gy (%)	Control group	2.62	2.62	0.617
	Enlargement group	2.95	3.06	
Parotid gland I_V30 Gy (%)	Control group	57.22	15.64	0.613
	Enlargement group	55.48	14.30	
Parotid gland C_V30 Gy (%)	Control group	54.44	14.23	0.432
	Enlargement group	52.09	11.96	

Abbreviation: C, contralateral; *D*_95_, the minimum dose delivered to 95% of the target; *D*_max_, maximum dose; I, ipsilateral; PSM, propensity score matching; V30 Gy, the relative volume of the structure receiving over 30 Gy; V60 Gy, the relative volume of the structure receiving over 60 Gy.

### 3.3 Univariate and multivariate analyses of dosimetric parameters

To further confirm the association between the tumor volume enlargement and subsequent radiotherapy, univariate and multivariate analyses were conducted to identify factors independently associated with the dosimetric parameters among all of the enrolled patients. As shown in Table 5, the tumor volume change of GTVnx (enlargement group vs. control group) and tumor volume change of GTVnd (enlargement group vs. control group) were not independently associated with any of the dosimetric parameters of PTVs and OARs.

**TABLE 5:** Univariate and multivariate analyses of dosimetric parameters

	T stage		N stage		Pretreatment GTVnx volume		Pretreatment GTVnd volume		Plan type		GTVnx volume change		GTVnd volume change	
	*B*	*p*	*B*	*p*	*B*	*p*	*B*	*p*	*B*	*p*	*B*	*p*	*B*	*p*
**Univariate analysis (N = 240)**														
PGTVnx_D95	−28.9	0.000	28.2	0.001	−1.0	0.000	0.6	0.023	19.0	0.245	10.2	0.682	−33.4	0.027
PGTVnd_D95	2.0	0.003	−21.9	0.001	0.6	0.001	−0.7	0.000	33.4	0.007	2.4	0.890	16.2	0.188
PTV1_D95	12.3	0.057	11.1	0.122	0.5	0.015	0.5	0.018	−26.1	0.065	−22.1	0.245	−11.3	0.424
PTV2_D95	8.0	0.389	36.5	0.000	0.5	0.119	1.2	0.000	79.1	0.000	10.7	0.682	8.5	0.666
Spinal cord_Dmax	13.7	0.562	42.2	0.105	1.8	0.015	1.5	0.062	−266.7	0.000	−56.2	0.462	−40.1	0.449
Spinal cord PRV_Dmax	95.6	0.002	26.8	0.431	5.4	0.000	1.6	0.138	−117.5	0.080	−64.7	0.512	21.7	0.756
Brainstem_Dmax	62.6	0.000	−1.7	0.912	2.2	0.000	0.1	0.857	−34.9	0.254	−3.3	0.939	21.7	0.503
Brainstem PRV_Dmax	115.3	0.000	−35.2	0.070	3.7	0.000	−0.6	0.318	5.7	0.882	−13.2	0.813	80.4	0.052
Optic chiasm_Dmax	933.8	0.000	−437.7	0.000	24.8	0.000	−8.9	0.003	831.5	0.000	67.1	0.798	380.5	0.057
Optic nerve I_Dmax	706.2	0.000	−345.8	0.000	20.8	0.000	−7.3	0.006	832.6	0.000	−2.9	0.990	337.1	0.057
Optic nerve C_Dmax	536.3	0.000	−216.4	0.007	16.3	0.000	−4.9	0.050	959.6	0.000	26.7	0.909	222.4	0.183
Lens PRV I_Dmax	219.6	0.000	−54.7	0.310	10.3	0.000	−3.3	0.047	−23.8	0.824	−128.2	0.420	20.9	0.804
Lens PRV C_Dmax	92.7	0.000	−45.1	0.007	3.3	0.000	−1.2	0.019	−117.8	0.000	−31.3	0.472	−19.0	0.596
Pituitary_Dmax	609.1	0.000	−270.2	0.000	17.3	0.000	−5.8	0.005	292.9	0.030	−68.9	0.708	302.0	0.030
Temporal lobe I_V60 Gy	4.5	0.000	−1.7	0.006	0.1	0.000	0.0	0.010	2.2	0.067	0.1	0.949	2.9	0.007
Temporal lobe C_V60 Gy	1.4	0.000	−0.2	0.655	0.1	0.000	0.0	0.184	1.1	0.111	−0.5	0.615	1.1	0.044
Parotid gland I_V30 Gy	1.1	0.355	0.6	0.657	0.1	0.024	0.1	0.024	−13.2	0.000	−2.0	0.610	−3.3	0.220
Parotid gland C_V30 Gy	−1.0	0.373	0.0	0.987	0.0	0.760	0.0	0.443	−15.8	0.000	−0.7	0.856	−2.6	0.276
**Multivariate analysis (N = 240)**														
PGTVnx_D95	−11.6	0.241	1.9	0.864	−0.5	0.126	0.3	0.299	—	—	—	—	−20.1	0.202
PGTVnd_D95	2.8	0.701	−8.1	0.317	0.5	0.044	−0.5	0.019	30.7	0.010	—	—	—	—
PTV1_D95	8.1	0.329	—	—	0.4	0.145	0.6	0.008	−26.2	0.059	—	—	—	—
PTV2_D95	—	—	20.5	0.087	—	—	0.9	0.020	81.7	0.000	—	—	—	—
Spinal cord_Dmax	—	—	—	—	2.0	0.005	1.5	0.047	−263.9	0.000	—	—	—	—
Spinal cord PRV_Dmax	−15.1	0.689	—	—	5.8	0.000	—	—	−118.8	0.061	—	—	—	—
Brainstem_Dmax	32.3	0.063	—	—	1.5	0.007	—	—	—	—	—	—	—	—
Brainstem PRV_Dmax	70.8	0.004	23.3	0.312	3.0	0.000	—	—	—	—	—	—	52.2	0.171
Optic chiasm_Dmax	608.7	0.000	55.4	0.601	11.6	0.000	−3.5	0.235	820.1	0.000	—	—	11.2	0.941
Optic nerve I_Dmax	327.5	0.000	−16.2	0.874	13.4	0.000	−3.0	0.292	802.0	0.000	—	—	−7.9	0.957
Optic nerve C_Dmax	297.0	0.000	−13.2	0.873	9.8	0.000	−1.7	0.483	895.2	0.000	—	—	—	—
Lens PRV I_Dmax	14.4	0.805	—	—	9.8	0.000	−2.4	0.117	—	—	—	—	—	—
Lens PRV C_Dmax	48.8	0.006	4.5	0.809	2.3	0.000	−0.9	0.088	−130.7	0.000	—	—	—	—
Pituitary_Dmax	382.4	0.000	74.0	0.360	9.6	0.000	−2.5	0.251	211.8	0.075	—	—	97.9	0.396
Temporal lobe I_V60 Gy	2.4	0.000	−0.7	0.267	0.1	0.000	0.0	0.445	1.5	0.116	—	—	1.0	0.274
Temporal lobe C_V60 Gy	0.4	0.255	—	—	0.0	0.001	—	—	—	—	—	—	0.7	0.164
Parotid gland I_V30 Gy	—	—	—	—	0.1	0.008	0.1	0.018	−13.0	0.000	—	—	—	—
Parotid gland C_V30 Gy	—	—	—	—	—	—	—	—	—	—	—	—	—	—

Abbreviations: C, contralateral; *D*_95_, the minimum dose delivered to 95% of the target; *D*_max_, maximum dose; I, ipsilateral; V30 Gy, the relative volume of the structure receiving over 30 Gy; V60 Gy, the relative volume of the structure receiving over 60 Gy.

## 4 DISCUSSION

Due to the complicated anatomical location, the tumor size of NPC has a significant influence on the dosimetric parameters of radiotherapy.^12^, ^13^, ^14^ Tumor volume enlargement after IC has been observed in a small proportion of NPC patients despite the high chemotherapy sensitivity of the cancer,^6^ but its influence on the subsequent radiotherapy plan has not yet been investigated. To the best of our knowledge, the current study is the first to address this problem. We compared the dosimetric parameters between patients with tumor volume enlargement and patients with tumor volume reduction after IC, and PSM was adopted to control the balance of other factors, including T stage, N stage, pretreatment GTVnx volume, pretreatment GTVnd volume, and plan type (tomotherapy vs. conventional IMRT).^15^, ^16^, ^17^, ^18^, ^19^, ^20^

Our results showed that GTVnx enlargement after IC had no significant impact on most of the dosimetric parameters. This finding is unexpected because it is expected that the primary tumor of NPC is closely related to the dosimetry of PTVs and OARs. A possible explanation for this phenomenon is attributed to the method of delineating the final GTVnx after IC, which was the summation of pre‐IC and post‐IC GTVnx according the recommendation of the international guidelines.^11^ Despite the difference in tumor volume change between the enlargement group and its matched control group, the difference in the final GTVnx was not statistically significant (47.5 cm^3^ vs. 40.1 cm^3^, *p* = 0.484). Additionally, the only disadvantage of the enlargement group was the protection of the contralateral lens PRV (Dmax, 722 cGy vs. 634 cGy, *p* = 0.041), which would not have significant influence on clinical outcomes because adherence to the dose limit of lens PRV (Dmax <900 cGy) was performed for both groups. Therefore, a GTVnx enlargement of ≥10% after IC has no significant influence on subsequent radiotherapy when the final GTVnx is defined as the summation of pre‐IC and post‐IC GTVnx.

It is worth mentioning that several studies have investigated the feasibility of using the post‐IC GTVnx as the final GTVnx. A randomized controlled study by Yang et al. showed that using the post‐IC GTVnx as the final GTVnx did not reduce the local control and survival rate in locally advanced NPC, but the doses to OARs decreased, and the quality of life improved.^21^ Another study by Xue et al. also indicated that contouring GTVnx based on the post‐IC images achieved satisfactory survival outcome and avoided overdosing of critical neurological structures.^22^ Similar results have been reported by several other studies.^23^, ^24^ If the post‐IC GTVnx is adopted as the final GTVnx in future practice, the potential influence of tumor volume enlargement on subsequent radiotherapy should not be ignored, as the volume of the final GTVnx between the enlargement group and the reduction group would be significantly different.

Our results also showed that GTVnd enlargement after IC had no significant impact on the dosimetric parameters of subsequent radiotherapy. It is noteworthy that the final GTVnd was defined as the post‐IC GTVnd only, and there was a significant difference in the final GTVnd between the enlargement group and its matched control group (18.2 cm^3^ vs. 8.1 cm^3^, *p* = 0.017). This insignificant influence of GTVnd enlargement can be attributed to the anatomical location of lymph nodes, which are not adjacent to the majority of the OARs, in most cases. Despite a GTVnd volume enlargement, the dose coverage of PTVs and the protection of OARs can be easily satisfied for most patients with modern radiotherapy techniques, such as the conventional IMRT and tomotherapy. This is supported by the results of the multivariate analysis of dosimetric parameters (Table 5), which indicated that N stage and pretreatment GTVnd volume were not independently associated with the dosimetry of almost all of the OARs. Similar results have also been reported by the study of Yao et al., which analyzed the radiation doses to OARs in 148 NPC patients and showed that N stage was not independently associated with the dosimetry of most OARs.^25^ Therefore, a GTVnd enlargement of ≥10% after IC has no significant impact on subsequent radiotherapy.

It should be noted that univariate and multivariate analyses of dosimetric parameters were also performed in the current study. As shown in Table 5, the volume changes of GTVnx and GTVnd after induction chemotherapy (enlargement group vs. control group) were not independently associated with any of the dosimetric parameters of PTVs and OARs, which is consistent with the results discussed above. In addition, the multivariate analysis indicated that T stage, pretreatment GTVnx volume, and plan type were independently associated with the parameters of most OARs, which is in accordance with the results of previous studies.^15^, ^16^, ^18^, ^19^, ^20^

Although PSM was adopted in our study to control the balance between the enlargement group and the control group, it should be noted that there were still some uncontrolled biases. First, one case in the GTVnx enlargement group and five cases in GTVnd enlargement group were discarded due to the lack of matched case in the control group. Second, some important factors may be not included in the process of matching, such as the distance between the tumor and the OARs, as there was no practical method which can provide such information. Third, cases with larger tumors may be excluded during the matching process, because the pre‐IC tumor volumes in the enlargement group were smaller than the control group before matching as shown in Table 1. It is worth mentioning that the larger pre‐IC tumor volumes in the control group indicates that larger tumors may be more sensitive to chemotherapy. Similar finding has been reported in the study of Wang et al., which showed that a larger tumor volume was independently associated with a higher likelihood of response to induction chemotherapy in head and neck cancer patients.^26^ A possible explanation for this phenomenon is that larger tumors are more likely to be involved with abundant blood supply,^27^, ^28^ resulting in higher concentration of chemotherapy drugs in the tumor tissues and better treatment responses.^29^, ^30^

The results of this study were potentially affected by several factors. First, 10% was adopted as the cut‐off value to determine tumor volume enlargement in our study. A higher cutoff value would significantly reduce the number of cases available for the propensity matching (especially for GTVnx) as shown in the supplement Table 1, which depicts the distribution of the relative volume change of GTVnx and GTVnd after IC. Second, the current study did not analyze the potential influence of the chemotherapy regimen, as docetaxel plus cisplatin was the only IC regimen administered at our center. Third, the survival outcomes were not analyzed in our study because the follow‐up time (median follow‐up time: 21 months) was too short to analyze the survival outcome of non‐metastatic NPC, which has a 5‐year OS of 70‐90%.^4^, ^31^ Last, the sample size of our research was small (only 20 pairs of matched patients for GTVnx and 39 pairs of matched patients for GTVnd), which should be taken into consideration while interpreting the results.

To summarize, a tumor volume enlargement of ≥10% in GTVnx or GTVnd after IC has no significant impact on the dosimetric parameters of subsequent radiotherapy in locally advanced NPC.

## ETHICS APPROVAL

This study was approved by the Ethics Committee of the Xiangya Hospital of Central South University prior to commencement.

## CONFLICT OF INTEREST

The authors declare that they have no conflict of interest.

## AUTHOR CONTRIBUTION

Liangfang Shen conceived and designed the analysis. Shan Li collected the data and performed the analysis. Liangfang Shen and Shan Li wrote the paper.

## Data Availability

The data that support the findings of this study are openly available in Mendeley Data at http://dx.doi.org/10.17632/gcg4j9y7cw.1
